# In Silico Target Identification of Galangin, as an Herbal Flavonoid against Cholangiocarcinoma

**DOI:** 10.3390/molecules27144664

**Published:** 2022-07-21

**Authors:** Brinda Balasubramanian, Simran Venkatraman, Kyaw Zwar Myint, Sucheewin Krobthong, Patompon Wongtrakoongate, Jittiyawadee Sripa, Panthip Rattanasinganchan, Pornphimon Metheenukul, Rutaiwan Tohtong

**Affiliations:** 1 Department of Biochemistry, Faculty of Science, Mahidol University, Bangkok 10400, Thailand; balasubramanian.bri@student.mahidol.edu (B.B.); patompon.won@mahidol.ac.th (P.W.); 2 Graduate Program in Molecular Medicine, Faculty of Science, Mahidol University, Bangkok 10400, Thailand; simran.ven@student.mahidol.ac.th (S.V.); kyawzwar.myn@student.mahidol.ac.th (K.Z.M.); 3 Center for Neuroscience, Faculty of Science, Mahidol University, Bangkok 10400, Thailand; sucheewin82@gmail.com; 4 College of Medicine and Public Health, Ubon Ratchathani University, Ubon Ratchathani 34190, Thailand; jittiyawadee.s@ubu.ac.th; 5 Faculty of Medical Technology, Huachiew Chalermprakiet University, Samut Prakan 10540, Thailand; panthip@hcu.ac.th; 6 Department of Veterinary Technology, Faculty of Veterinary Technology, Kasetsart University, Bangkok 10900, Thailand; pornphimon.m@ku.th

**Keywords:** Galangin, molecular docking, proteomics, cholangiocarcinoma, anti-cancer

## Abstract

Cholangiocarcinoma (CCA) is a heterogenous group of malignancies in the bile duct, which proliferates aggressively. CCA is highly prevalent in Northeastern Thailand wherein it is associated with liver fluke infection, or *Opisthorchis viverrini* (OV). Most patients are diagnosed in advanced stages, when the cancer has metastasized or severely progressed, thereby limiting treatment options. Several studies investigate the effect of traditional Thai medicinal plants that may be potential therapeutic options in combating CCA. Galangin is one such herbal flavonoid that has medicinal properties and exhibits anti-tumor properties in various cancers. In this study, we investigate the role of Galangin in inhibiting cell proliferation, invasion, and migration in OV-infected CCA cell lines. We discovered that Galangin reduced cell viability and colony formation by inducing apoptosis in CCA cell lines in a dose-dependent manner. Further, Galangin also effectively inhibited invasion and migration in OV-infected CCA cells by reduction of MMP2 and MMP9 enzymatic activity. Additionally, using proteomics, we identified proteins affected post-treatment with Galangin. Enrichment analysis revealed that several kinase pathways were affected by Galangin, and the signature corroborated with that of small molecule kinase inhibitors. Hence, we identified putative targets of Galangin using an in silico approach which highlighted c-Met as candidate target. Galangin effectively inhibited c-Met phosphorylation and subsequent signaling in in vitro CCA cells. In addition, Galangin was able to inhibit HGF, a mediator of c-Met signaling, by suppressing HGF-stimulated invasion, as well as migration and MMP9 activity. This shows that Galangin can be a useful anti-metastatic therapeutic strategy in a subtype of CCA patients.

## 1. Introduction

Cholangiocarcinoma (CCA) is an aggressive group of malignancies in the bile duct epithelium. This cancer is highly prevalent in the Northeastern regions of Thailand, with incidences climbing worldwide. The cancer is highly heterogenous in nature, such that therapeutics approved in the west for CCA prove inefficacious in Thai tumors. This is due to the concordance of most Thai CCA patients presenting with a liver fluke infection, *Opisthorchis viverrini*, which is followed by chronic inflammation and CCA tumorigenesis [1]. Currently, there are poor diagnostic systems to detect the cancer at earlier stages, ensuing in a growing number of patients diagnosed in more advanced stages. At later stages, the cancer belligerently metastasizes thus eliminating the option for surgical resection. The survival median of unresectable CCA patients is less than 12 months, whereas the 5-year survival rate of patients who avail curative resection is between 0–40%. Moreover, three-year survival rates of 35 to 50% is seldom achieved in CCA patients when negative histological margins are attained during surgery [2,3]. Even patients who can avail surgical resection exhibit poor prognosis and frequent tumor relapses [4]. Alternatively, patients in advanced stages who receive standard-of-care chemotherapy or palliative care either fail to respond to treatment, experience side-effects and or risk the chance of cancer recurrence. Hence, these limited treatment options compel the need for novel therapeutic methods. 

The aggressive nature of CCA is a result of several contributing factors, including chronic inflammation of the bile duct epithelium, biliary irritation, and parasitic infection. One such factor is the bi-directional interplay of the tumor microenvironment (TME) and CCA cells. The tumor microenvironment is constituted of neoplastic epithelial cells, stromal cells, and the extracellular matrix (ECM). TME plays a vital role in driving CCA progression, migration, and invasion [5]. Current investigations reveal that traditional Thai medicinal plants exhibit various bioactivities, including anti-bacterial, anti-fungal [6], anti-oxidant, anti-inflammatory [7], and anti-tumor activity [8]. This shows promise for using Thai herbs as an alternative agent for CCA treatment. One such medicinal herb is Alpinia galanga (Linn.) Sw. or galangal, a culinary herb from the family Zingiberaceae, which is widely used in various Asian countries. One of the major bioactive compounds of A. galanga is Galangin or 3,5,7 trihydroxyflavone, a polyphenolic compound [9]. Galangin has shown anti-tumor potential in various cancers, which is owed to its anti-genotoxic, anti-mutagenic, and anti-oxidative characteristics. Most flavonoid compounds including Galangin are known to inhibit cell proliferation through the inhibition of tyrosine-specific kinases or through the induction of apoptosis-related genes [9]. Moreover, flavonoids are now garnering interest for inhibition of receptor tyrosine kinases (RTKs) in several cancer types [10,11]. It is well known that RTKs are frequently dysregulated in CCA [12]. Hence, we hypothesized that Galangin may elicit its anti-tumor potential by inhibiting essential kinases in CCA. In this study, we developed an in integrated in silico approach to identify the targets of Galangin and thereby unravel the underlying mechanism of its inhibition of cancer progression in CCA. Here we show that Galangin exhibits anti-metastatic properties in CCA by reducing MMP9 activity via the inhibition of c-Met phosphorylation.

## 2. Results

### 2.1. Galangin Inhibits Cell Proliferation in CCA Cells via Induction of Apoptosis

**Figure 1:**
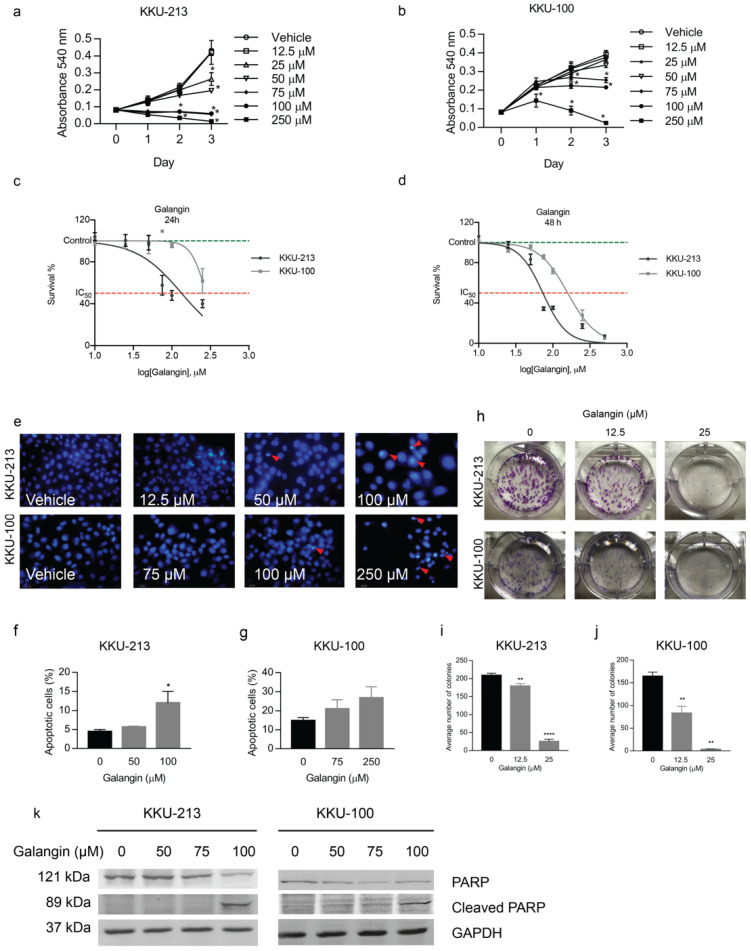
Effect of Galangin on cholangiocarcinoma cell proliferation. The cytotoxic effect of Galangin on (**a**) KKU-213, (**b**) KKU-100, and CCA cell lines were determined by MTT assay. The IC_50_ values were calculated using the dose-response curve for (**c**) 24 h and (**d**) 48 h of treatment. (**e**) Detection of apoptosis by DAPI staining after Galangin treatment in CCA cell lines in various concentrations. The number of apoptotic cells were quantified in (**f**) KKU-213 and (**g**) KKU-100 cell lines through FACS analysis. (**h**) Representative images of colony formation and quantification for average number of colonies on (**i**) KKU-213 and (**j**) KKU-100 cells upon Galangin treatment at 10 and 25 µM. (**k**) Effect of Galangin on protein involved in apoptosis. Cellular proteins were analyzed by Western blot assay for the expression of PARP and cleaved PARP. Values represents the mean ± SEM of three independent experiments, * *p* ≤ 0.05, ** *p* ≤ 0.01, **** *p* ≤ 0.0001 compared with the untreated control.

Galangin exhibited cytotoxic activity in all the tested CCA cells, at high concentrations (75–250 μM) by inhibiting cell proliferation and colony-formation in a dose-dependent manner. KKU-213 cells were the most sensitive to Galangin treatment (IC_50_~134 ± 1.2, 73 ± 1.1 µM at 24 and 48 h, respectively), whereas KKU-100 cells were the least sensitive (IC_50_~279 ± 1.2, 158 ± 1.0 µM at 24 and 48 h, respectively) when compared to other CCA cell lines (Figure 1a–d). Previously, Galangin has been reported to induce apoptosis in CCA, therefore, to confirm that this phenomenon is maintained in our system we performed DAPI staining and Annexin V and PI staining to quantify apoptotic cells. We found that Galangin increased apoptotic nuclei, characterized by condensed chromatin as opposed to a more relaxed chromatin in non-apoptotic cells, at 24 h of treatment (Figure 1e) in KKU-213 and KKU-100 cells. Furthermore, Annexin V and PI staining of CCA cell lines upon Galangin treatment revealed a significant increase in apoptotic cells at 100 uM and 250 uM compared to untreated control in KKU-213 and KKU-100 cells, respectively (Figure 1f,g).

To assess delayed cytotoxicity of CCA cell lines to Galangin, we conducted a colony formation assay. Galangin significantly inhibited the colony formation of KKU-213 (Figure 1h,i, *p* = 0.0035, 0.0001) and KKU-100 cells (Figure 1h,j, *p* = 0.0086, 0.0059) in a dose dependent manner. In addition, Galangin was able to lead the cells to terminal stages of apoptosis as evidenced by decreased full-length PARP expression and subsequent increase in cleavage of PARP in both CCA cells (Figure 1k). Together, these results demonstrate that Galangin induces apoptotic cell death in CCA cells at high concentrations. 

### 2.2. Galangin Inhibits Metastatic Phenotypes of CCA Cells

**Figure 2:**
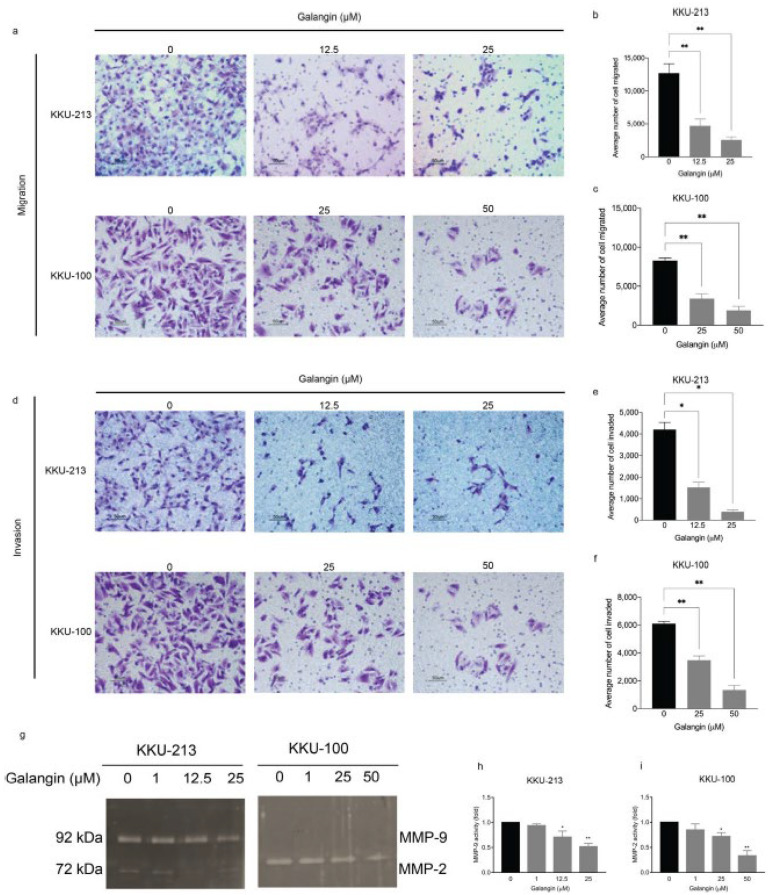
Effect of Galangin on migration and invasion in KKU-213 and KKU-100 CCA cells. Transwell assay was used to detect cell migration and invasion. Images were taken at 20× magnification. Images of (**a**) cell migration, each cell group treated with various of Galangin, and their respective quantifications in (**b**) KKU-213 and (**c**) KKU-100 cells. Images of (**d**) cell invasion showing each cell group treated with various amounts of Galangin, and their respective quantifications in (**e**) KKU-213 and (**f**) KKU-100 cells. (**g**) Representative images of the gelatin zymography and (**h**) quantification the band intensity of MMP-9 activity in KKU-213 and (**i**) quantification of the band intensity of MMP-2 activity in KKU-100 after Galangin treatment in various concentrations. * *p* ≤ 0.05, ** *p* ≤ 0.01.

As previously stated, Galangin has displayed anti-metastatic properties in other cancers [13], hence, we hypothesized that these effects are maintained in CCA. We explored the effect of Galangin on the preparatory steps (cell invasion and migration) towards metastasis in CCA cell lines. In order to avoid the false-positive result of reduced migration and invasion due to cytotoxicity, we used lower, non-lethal doses. We found that, post-treatment with Galangin, there was a significant reduction in cell migration in KKU-213 (Figure 2a,b, *p* = 0.0062, 0.0090) and KKU-100 (Figure 2a,c, *p* = 0.0071, 0.0036) when compared with vehicle treated cells. Additionally, Galangin also decreased the number of invading cells in KKU-213 (Figure 2d,e, *p* = 0.0424, 0.0131) and KKU-100 (Figure 2d,f, *p* = 0.0065, 0.0038) when compared with vehicle treated cells in a dose-dependent manner. To elucidate the underlying mechanism as to how Galangin exudes the observed anti-metastatic phenotypes (cell invasion and migration), we assessed the effect of Galangin on matrix metalloproteinase (MMPs) activity. Proteolytic enzyme MMP2 and MMP9 facilitate cell invasion and migration by degrading the extracellular matrix (ECM), thereby allowing the invasion of tumor cells into adjacent tissues and the subsequent migration of cells into circulation [14,15]. Increasing concentrations of Galangin significantly reduced MMP9 and MMP2 activity in a dose-dependent manner in both KKU-213 (Figure 2g,h, *p* = 0.0376, 0.0038) and KKU-100 (Figure 2g,i, *p* = 0.0476, 0.0022), respectively, when compared with untreated control. It is noteworthy that KKU-213 cells predominantly secrete MMP9, whereas KKU-100 secretes MMP2 (Figure 2g). These results confirm our hypothesis that Galangin exudes anti-metastatic phenotypes by the reduction of cell invasion and migration by inhibition of MMP activity in CCA.

### 2.3. Proteomics Analysis Reveals Affected Proteins Post Treatment with Galangin in CCA

**Figure 3:**
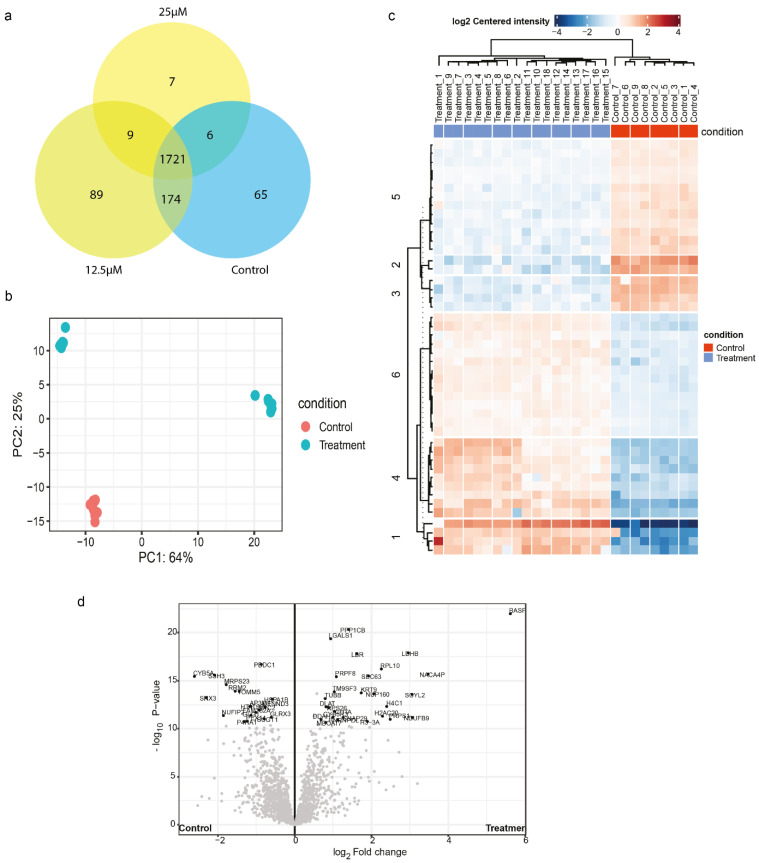
Proteomics analysis upon Galangin treatment in KKU-213 cells. (**a**) Venn Diagram of uniquely identified peptides in each condition. (**b**) Principle-component analysis of control and Galangin treated samples. (**c**) Heatmap of differentially expressed proteins between treatment and control with the significance criteria of proteomics fold change at log2 (1.5) and *p* < 0.05. (**d**) Volcano plot depicting the differentially expressed proteins (DEP) between control and treatment samples.

To better understand the molecular pathways affected by Galangin treatment in CCA, we performed LC-MS using Orbitrap HF hybrid mass spectrometer combined with an EASY-nLC1000 nano-liquid chromatography. As a measure of quality control, proteomics data were obtained using the Orbitrap mass spectrometry and resulted in a total of 2450 proteins. The full proteome spectra were processed using Proteome Discoverer 2.4 and were identified using the Uniprot database. The proteome identification is provided in Supplemental Table S1. The mass deviation analysis revealed 92.51% of identified peptide groups had ≤ 10 ppm suggesting that the major peptides achieved a good mass precision. The narrow peptide mass deviation ensures the reduction in the number of false-positive peptides. Although a few miscleavage sites of tryptic peptide are a common phenomenon in the proteomics analysis, we observed 90.30% of all identified peptides had no miscleavage site and 9.27% had a miscleavage site. We determined the unique peptides per condition and commonalities with other conditions (Figure 3a). Overall, there were 1721 common peptides. Amongst treated samples (25 μM and 12.5 μM), there were 9 common peptides, which were annotated as such, serine-threonine protein kinase, core-histone macro-H2A, glycolipid transfer protein, and brain acid soluble protein 1. It is seen that treatment at 12.5 μM yielded more unique peptides, compared to control and 25 μM treatment. When assessing the proteomic differences between treatment and control, using PC analysis, we noticed that the treated samples represent distinctly different populations compared to control (Figure 3b). Furthermore, we explored the differentially expressed proteins using the ‘DEP’ workflow from Bioconductor. We grouped samples of both treatment conditions and comparing the differentially expressed proteins with control, we observed 46 differentially expressed proteins, of which, 18 proteins were downregulated upon treatment, and 28 proteins were upregulated upon treatment, when compared to control as represented in the heatmap and volcano plot. The upregulated proteins included: LGALS1, KRT9, NUP160, SNAP29. On the other hand, the downregulated proteins included: PBDC1, GLG1, RRM2, CYB5A, SNX3, and SSH3 (Figure 3c,d, Supplementary Table S2). 

### 2.4. Enrichment Analysis of Affected Proteins Belong to Several Kinase Signalling Pathways and Can Be Targeted by Small Molecule Kinase Inhibitors

**Figure 4:**
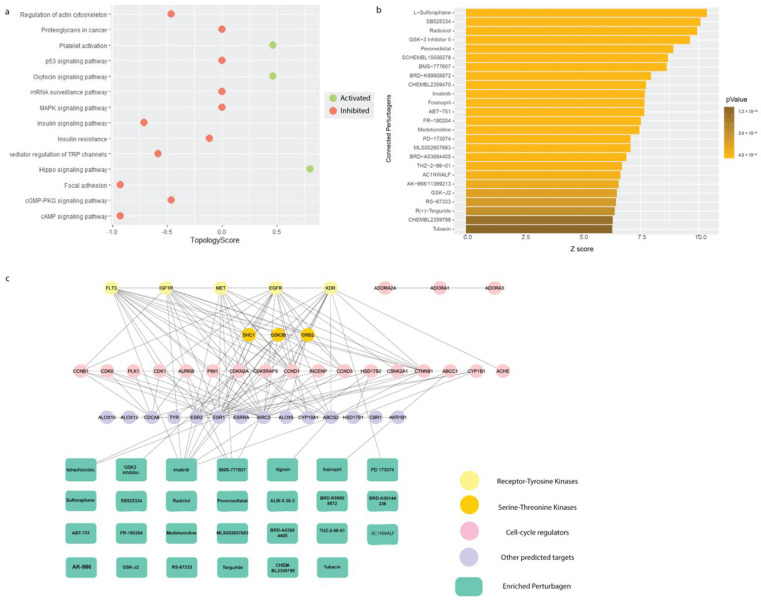
Pathway enrichment and pharmaco-connectivity analysis of the DEP signature. (**a**) Signaling pathway impact analysis (SPIA) of the DEP signature. Green nodes represent pathways that are activated (topology score > 0), and red nodes represent pathways that are inhibited (topology score < 0). (**b**) Significant positively connected perturbagens to the DEP signature, ranked by lowest *p*-value and highest Z-score. (**c**) Pharmaco-connectivity analysis between the positively connected perturbagens and the predicted targets of Galangin based on structural similarity.

Using these differentially expressed proteins, we assessed the pathways affected by Galangin treatment using signaling pathway impact analysis (SPIA) from the iLINCs tool. We found that MAPK pathway, focal adhesion, cAMP signaling, insulin signaling, and actin cytoskeletal regulation were inhibited, whereas pathways such as platelet activation, hippo signaling pathway, and oxytocin signaling pathway were activated (Figure 4a). Using the same input to enrich connected small molecules that may have the potential to reverse this proteomic signature from the LINCS pharmaco-connectivity tool, we found several RTK inhibitors that were significantly enriched (Figure 4b). Taken together, the differentially expressed proteome analysis was indicative of the role of Galangin in inhibiting RTK signaling.

### 2.5. In Silico Target Prediction Which Identified a Number of Kinases as Potential Targets of Galangin

We also employed in silico target prediction based on structural similarity using the SMILES structure of Galangin using SwissTargetPrediction (Supplementary Table S4). We see that amongst the predicted targets, the class of kinases makes up 31% of the predictions. This corroborates our finding that positively correlated perturbagens that mimic the proteomic profile elicited upon Galangin treatment consist of kinase inhibitors. This provided the rationale to further investigate the ability of Galangin to inhibit tyrosine kinase signaling. Further, we assessed the pharmaco-connectivity between the predicted targets and the positively correlated perturbagens using STITCH-DB. We found that several predicted inhibitors including imatinib, BMS 777607, and PD-173074 targeted the predicted targets of Galangin (Figure 4c). 

**Figure 5:**
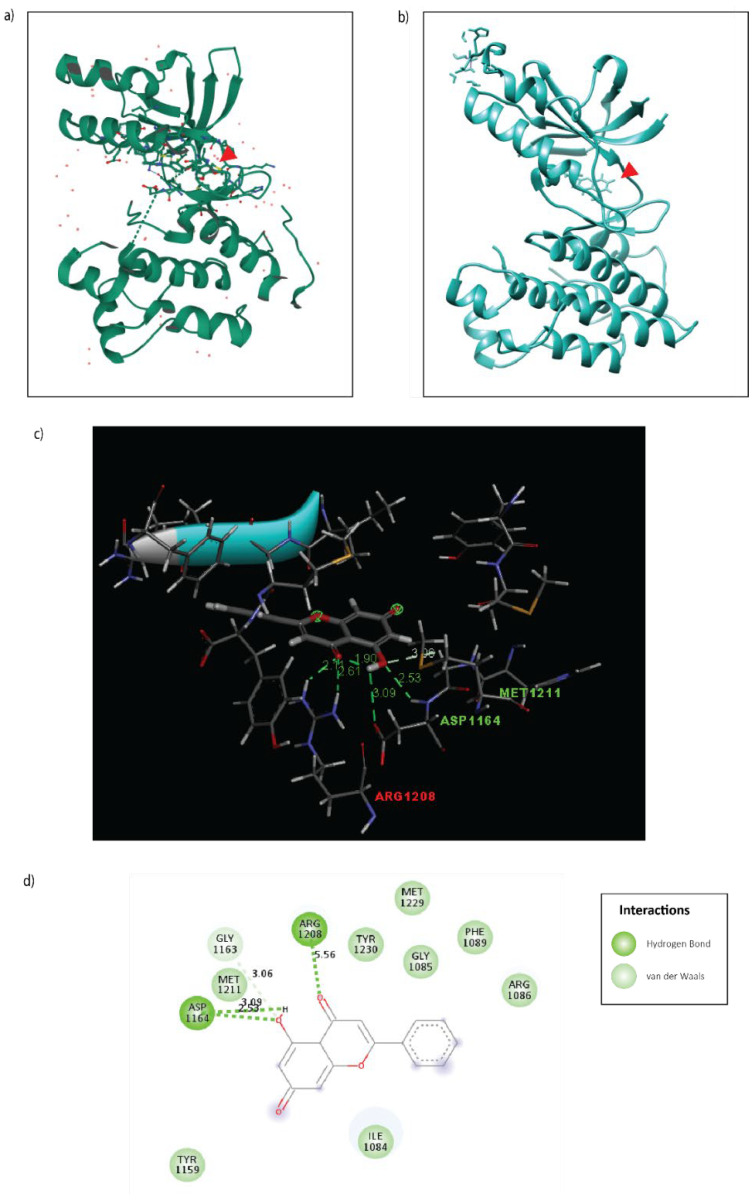
Molecular docking of Galangin to the tyrosine kinase domain of the c-Met receptor. (**a**) 3D X-ray crystallography structure of c-Met inhibitor, AM7, bound to c-Met (PDB: 2RFN). (**b**) 3D Docking model of Galangin (PDB: 57D docked to the c-Met receptor (PDB:1R1W) using SwissDock. (**c**) 3D interaction plot of Galangin binding to c-Met receptor with distance in (Å). (**d**) 2D interaction plot of Galangin binding to c-Met receptor. The important interactions were highlighted, including conventional hydrogen bond (dark green dot line) and van der Waals (light green).

Of the several predicted targets based on structural similarity, c-Met was considered for further validation as it corroborated the biological phenotypes observed in CCA upon Galangin treatment. c-Met is a key driver of cholangiocarcinoma progression with more than half of biliary tumors displaying an overexpression of the receptor [16]. Hence, to validate this predicted target, we explored the probable binding affinity between the ligand, Galangin, and the receptor c-Met by employing molecular docking. Galangin (PDB: 57D) was molecularly docked with the previously established crystallized structure of tyrosine kinase domain of the c-Metreceptor (PDB: 1R1W) using SwissDock [17] (Figure 5). We compared the binding conformation to that of an X-ray crystallography structure of an established c-Met receptor inhibitor, AM7, to the tyrosine kinase domain of c-Met (PDB: 2RFN) (Figure 5a). We found that Galangin does bind to the tyrosine kinase domain in a similar manner of several conformations, with a predicted binding affinity of ΔG = −6.233 kcal/mol, and a full fitness of −1365.861 kcal/mol. This conformation is bound by hydrogen bonds with Asp-1164 with an estimated distance of 2.53–3.09 Å and Arg-1208 with a distance of 5.56 Å and van der Waals binding in residues including Met-1211 and Tyr-1230 (Figure 5b–d). 

### 2.6. Galangin Inhibits c-Met Signalling

**Figure 6:**
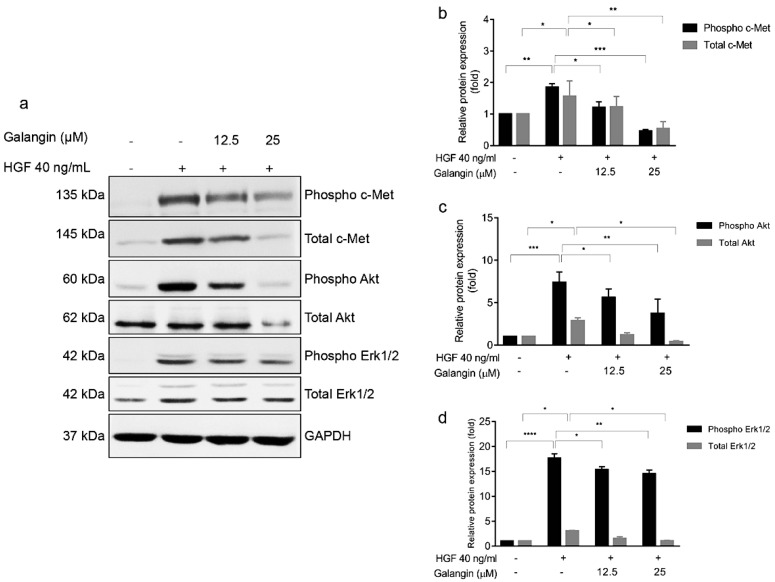
Galangin treatment with HGF-stimulated KKU-213 cells affects c-Met signaling. (**a**) Effect of Galangin on HGF-stimulated cell signaling by western blotting. Cells were analyzed for (**b**) phospho c-Met, total c-Met, (**c**) phospho Akt, total Akt, (**d**) phospho ERK1/2, and total ERK1/2 expression by Western blotting. GAPDH was used as loading control to normalize the amount of protein in each experiment conditions. Data are the mean ± SEM from three independent experiments, * *p* ≤ 0.05, ** *p* ≤ 0.01, *** *p* ≤ 0.001, **** *p* ≤ 0.0001 compared with the HGF treatment.

Our in silico analysis implicates c-Met as one of the direct targets of Galangin, hence, we hypothesized that Galangin would effectively inhibit the HGF/c-Met signaling cascade. Our results show that HGF was able to induce the phosphorylation of c-Met receptor and its subsequent signaling cascade, Akt and ERK(Figure 6a). Notably, we found that Galangin effectively inhibited HGF-induced phosphorylation of c-Met receptor and downregulated total c-Met expression in KKU-213 cells in a dose-dependent manner. Similarly, Galangin also suppressed HGF-induced phosphorylation Akt and ERK as well as total expression (Figure 6a). Therefore, we successfully validated our findings from the in silico analysis in in vitro system in CCA. 

### 2.7. Galangin Suppresses HGF-C-Met Axis Mediated Invasion and Migration of CCA Cells

**Figure 7:**
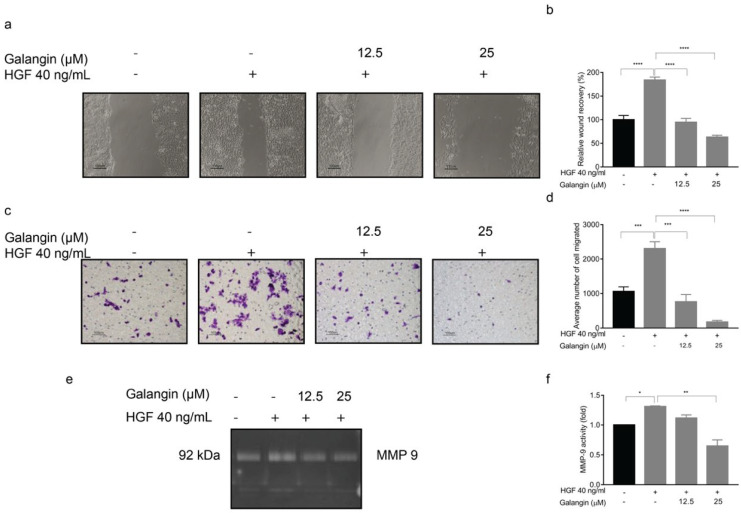
Effect of Galangin upon HGF-stimulation on wound-healing, cell migration, and MMP-9 activity in KKU-213 CCA cells. KKU-213 cells were stimulated with HGF and treated with various concentrations of Galangin. Representative images of (**a**) wound-healing and its (**b**) quantitation. Representative images of (**c**) Transwell cell migration and its respective (**d**) quantitation. Representative images of (**e**) MMP-9 activity and (**f**) its quantitation. Data are the mean ± SEM from three independent experiments, * *p* ≤ 0.05, ** *p* ≤ 0.01, *** *p* ≤ 0.001, **** *p* ≤ 0.0001 compared with the HGF treatment.

In line with our previous results, we wanted to evaluate if the anti-metastatic phenotypes exuded by Galangin were mediated via c-Met inhibition. The wound healing results indicated that Galangin significantly inhibited HGF-induced wound closure when compared with HGF treatment alone (Figure 7a,b, *p* ≤ 0.0001). This was consistent with chemoattractant migration assay by Transwell, wherein there was a reduction in the average number of migrated cells (Figure 7c,d). We then examined the MMP9 activity after stimulating KKU-213 with HGF using gelatin zymography. The results showed that HGF treatment was able to increase MMP9 activity, and Galangin significantly reduced the HGF-induced MMP9 activity (Figure 7e,f, *p* = 0.0081). Cumulatively, these results suggest that Galangin can suppress HGF-stimulated invasion and migration of CCA cells via the c-Met pathway.

## 3. Discussion

Due to the aggressive yet asymptomatic nature of CCA, most diagnoses occur after the cancer metastasizes and the disease has considerably progressed [18]. Treatment options that are the current gold-standard, such as chemotherapy, radiotherapy, and surgery have achieved little success in abating CCA progression. Therefore, the urgency of discovering novel therapies is pertinent to improve patient response and survival. Recently the use of traditional herbal compounds has shown promising anti-cancer activity. One of which is Galangin, a naturally active flavonoid from the root of *Alpinia officinarum* [19]. Galangin has been reported to have a variety of biological activities, including anti-tumor, anti-mutagenic, anti-oxidative, bactericidal, and anti-fibrotic effects. Our results reiterated that Galangin exhibits anti-proliferative and anti-metastatic activity in CCA cells. Here, we report for the first time using an in silico approach, that Galangin is able to inhibit the HGF/c-Met axis in CCA, which is implicated for its oncogenic role in several cancer types, including CCA [3,20].

Our results indicated that, at higher concentrations, Galangin induced apoptotic cell death in CCA cells as demonstrated through DAPI staining, Flow cytometry, and Western Blot analysis (Figure 1). Results from previous studies are consistent with our findings that Galangin inhibits cell growth in breasts, melanoma, and HCC, through apoptosis [21,22,23,24]. 

Therefore, Galangin, like other flavonoids, has proven to be a strong therapeutic agent to treat cancers by moderating apoptosis [25]. While the anti-proliferative effect of Galangin has been previously reported and our results also confirm this finding in different CCA cells, we report for the first time that CCA cells exhibit increased sensitivity to prolonged treatment at significantly lower concentration (Figure 1h). Moreover, we found that the anti-metastatic properties of Galangin in CCA, which includes cell invasion, migration, wound healing, and MMP activity, ensues at much lower concentrations than previously reported (Figure 2 and Figure 3). Finding the most efficacious dose and treatment duration is integral in translating the results from pre-clinical models to patients, as unreasonably high concentrations in in vitro studies may result in adverse drug response or toxicity in patients and also increase the risk of off-target activities [26]. Therefore, our results shed new light on Galangin’s role in inhibiting the progression of CCA. 

In addition to finding the most efficacious dose in limiting cancer cell proliferation and cell invasion and migration, the present study also proposed putative targets of Galangin to elucidate the underlying mechanism of action. We utilized an integrated in silico approach with a combination of proteomics, structural similarity target prediction, molecular docking and pharmaco-connectivity analysis followed by in vitro studies. This provided us molecular insight into the mode of action of Galangin to understand and corroborate the observed biological phenotypes. Our proteomics analysis showed that multiple pathways were inhibited following treatment with Galangin, including MAPK signaling, actin cytoskeletal regulation, and Focal adhesion (Figure 4). This corroborates our results where Galangin significantly suppressed cell invasion and migration. Moreover, this proteomics signature was positively correlated to that of several tyrosine kinase inhibitors, including c-Met inhibitors, imatinib, and BMS-777607. Lastly, we also identified various receptor tyrosine kinases such as MET, EGFR, IGF1R, FLT3, and KDR as putative targets of Galangin. These results suggest the potential signaling pathways by which anti-cancer effects may be executed by Galangin in CCA. 

Of these targets, the role of c-Met in CCA progression has been well characterized. In a prior investigation, it was seen that siRNA-specific targeting of c-Met in CCA cell lines resulted in suppression of proliferation, migration, and invasion of CCA cells when stimulated with HGF [3]. This is in line with our findings as we observed a similar effect on the biological phenotypes with Galangin treatment. Thus, we hypothesized that the biological mechanism of Galangin may be mediated through c-Met inhibition. This is confirmed by molecular docking of Galangin to the tyrosine kinase domain of the c-Met receptor, which is similar compared to the conformation of the X-ray crystallography structure of a c-Met inhibitor, AM7. Moreover, Galangin forms a hydrogen bond at Asp-1164 and several van der Waal’s interactions along Met-1211, Arg-1208, and Tyr-1230. These regions fall within the C-helix structure of the tyrosine kinase domain of the c-Met receptor [27]. This is akin to the span at which AM7 binds to c-Met. Hence, we postulated that Galangin inhibits the phosphorylation of c-Met in a similar manner.

CCA tissues were reported to have overexpression of c-Met and inhibition of HGF/c-Met pathway reduced the invasion of CCA cells [28,29]. We stimulated the CCA cells with HGF which, in turn, increased the phosphorylation of c-Met and subsequent signaling proteins, ERK and Akt (Figure 6a). In addition, HGF stimulation also increased cell migration, invasion, and MMP2 and MMP9 activity, respectively, in KKU-213 (Figure 7). The interplay between RTK signaling and MMPs has been well established. Several reports show that MMPs modulate the ECM to deliver ligands to their respective receptors and conversely, MMP expression is regulated by HGF/c-Met signaling, often mediated by Akt [30,31,32,33]. Our experiments thus far showed that Galangin inhibited invasion and migration of CCA cells via the inhibition of MMP9 activity. In addition, our results suggested that Galangin suppressed HGF-stimulated cell migration, invasion, and MMP2 and MMP9 activities in KKU-213 (Figure 7).

One of the limitations of this study is that we used in silico methods to predict targets, while our validatory experiments in in vitro models were largely focused on c-Met signaling, which was one among several kinases which may be potential targets of Galangin. Future investigative attention may be directed towards exploring other targets of Galangin to explore its potential multi-kinase inhibitor. Moreover, the predicted binding affinities of a putative conformation may not resemble experimental binding energies. However, the results obtained encourage future investigations to structurally validate these findings. 

Cumulatively, this study is the first scientific evidence of Galangin’s ability to exude anti-metastatic properties such as invasion and migration in the presence of HGF stimulation. In addition, our results demonstrate that Galangin was able to suppress c-Met phosphorylation suggesting that Galangin may exhibit its anti-metastatic activity via c-Met. Future investigations of Galangin should be directed towards elucidating its role in c-Met inhibition as well as other predicted targets of Galangin that we identified using an in silico approach in this study. While high concentrations of Galangin may induce apoptosis, we here showed at this herbal flavonoid has significant anti-metastatic activity at lower concentrations. Our findings suggest that Galangin can be used as an anti-progressive or anti-metastatic or prophylactic supplement. Aljarba et al., using in silico ADME-tox prediction studies, suggest that Galangin has good absorption properties orally with good tolerance. Moreover, they posit that Galangin can be distributed throughout the body intactly to exhibit desired effect [34]. However, further investigations are warranted to explore the potential use of Galangin as a preventative measure. 

Moreover, this is the first report to use an integrated computational approach to identify the underlying mechanism of Galangin in CCA. Here we showed that Galangin significantly reduced cell invasion and migration induced by HGF treatment. In addition, our results show that Galangin can inhibit MMP2 and MMP9 enzymatic activity in both HGF-stimulated and unstimulated conditions. Finally, our results illustrate that Galangin can inhibit HGF/c-Met axis in CCA. Thus proving that Galangin is potentially a bifunctional therapeutic agent for preventing cancer cell proliferation and disease progression. Future investigative attention should be directed to developing Galangin as a targeted therapy drug and exploring its potential in inhibiting tumorigenesis. 

## 4. Materials and Methods

### 4.1. Chemicals and Reagents

Galangin was purchased from Sigma Chemicals (Product No. 282200, Sigma-Aldrich, St. Louis, MO, USA). Recombinant human hepatocyte growth factor (rhHGF) was obtained from Immunotools (Cat N° 11343415, Friesoythe, Germany) and were resuspended in 0.1% BSA/PBS (AMRESCO, Solon, OH, USA) to 100 µg/mL stocks, aliquoted and stored at −80 °C. MTT was purchased from AppliChem (AppliChem Gm bH, Darmstadt, Germany). DAPI, Annexin-V, and Propidium Iodide (PI) were obtained from Invitrogen (Thermo Fisher Scientific, Massachusetts, MA, USA). The migration/invasion assay was done using Transwell chambers (Costar^®^, Corning, Kennebunk, ME, USA) with 6.5 mm diameter polycarbonate membranes (8 µm pore size). Primary antibodies against PARP (#9548), cleaved PARP (#5625), phospho c-Met (#3126), total c-Met (#3127), GAPDH (#47724) were purchased from Santa Cruz Biotechnology, Inc. (Dallas, TX, USA). Secondary antibodies (anti-mouse and anti-rabbit) were purchased from Cell Signaling Technology (Beverly, MA, USA).

### 4.2. Cell Culture

KKU-213 and KKU-100 human CCA cell lines, were purchased from the Japanese Collection of Research Bioresources Cell Bank, maintained in Ham’s F-12 culture medium supplemented with 10% FBS (Gibco, Life technologies, Grand Island, NY, USA) and incubated at 37 °C with 5% CO_2_. 

### 4.3. MTT Assay

5 × 10^3^ cells per well were seeded in a 96-well plate for 24 h and treated with various concentrations (0–250 µM) of Galangin. Cell viability was assessed at 24 h intervals up to 72 h post-treatment. Vehicle control was 0.1% DMSO-containing culture medium. MTT assay was performed according to standard protocol [35]. 

### 4.4. Clonogenic Assay

1 × 10^3^ cells per well were seeded in 6-well plates and incubated for 24 h. After incubation, cells were treated with various concentrations of Galangin in serum-free Ham’s F-12 culture medium for 24 h. Then, treatment medium was replaced by complete medium and incubated for 7 days. The colonies were fixed and then stained with 0.5% crystal violet and counted manually.

### 4.5. DAPI Staining

3 × 10^4^ CCA cells per well were seeded into a 24-well plate for 24 h and treated with various concentrations of Galangin for 48 h. After treatment, cells were washed with warm PBS and fixed with 4% paraformaldehyde +2% sucrose/PBS. Following which, the cells were stained with DAPI for 30 min and then washed with PBS 3 times. Cells were examined for nuclear condensation and fragmentation and photographed under fluorescence microscope.

### 4.6. Apoptosis Assay

3 × 10^5^ cells per well were seeded into a 6-well plate for 24 h and treated with various concentrations of Galangin for 24 h. Then, cells were harvested and washed twice with cold PBS. Cells were resuspended in Annexin V-binding buffer and stained with Annexin V and PI and then, incubated at room temperature for 5 min in the dark, before being analyzed with FACS Canto flow cytometer (BD Biosciences, Franklin Lakes, NJ, USA). The percentage of apoptotic cells is an addition of the percentage of early and late apoptotic cells.

### 4.7. Transwell Migration and Invasion Assay

1 × 10^4^ for KKU-213 and 8 × 10^4^ for KKU-100 were seeded, respectively, in the upper chamber of Transwell Boyden chambers. 200 µL of cell suspension containing varying concentrations of Galangin was added to the upper chamber. Sublethal concentrations of Galangin, 12.5 and 25 µM for KKU-213, and 25 and 50 µM for KKU-100 were used. Media supplemented with 10% FBS were used as a chemoattractant in the lower chamber. After 18 h of incubation at 37 °C, migrated cells were fixed with 30% methanol before staining with 4% (*w*/*v*) crystal violet. The insert chambers were washed with tap water, dried, and visualized at ×200 magnification for migrated cells. 

For invasion assay, the Transwell filters were coated with 0.4 µg/µL Matrigel (Corning, NY, USA) overnight on the day before seeding the cells. Cell suspensions were prepared in the presence or absence of 40 ng/mL HGF and/or Galangin (12.5 or 25 µM). Then, the cells are allowed to migrate for 6 h by incubating at 37 °C.

### 4.8. Gelatin Zymography

4 × 10^5^ cells per well were seeded into a 6-well plate. After 24 h of incubation in media containing 10% FBS, medium was replaced with 1 mL of serum-free medium containing Galangin (1, 12.5 and 25 µM for KKU-213 and 1, 25 and 50 µM for KKU-100). Supernatants were collected after 12 h incubation and stored at −80 °C. Then, 20 µL of conditioned media were used to evaluate the activity of MMP2 and MMP9. The conditioned media were mixed with loading buffer and the mixture was applied to 7% acrylamide gel containing 1% gelatin. After electrophoresis, SDS was removed by 2.5% Triton X-100 to renature the gelatinase. Gel was incubated in developing buffer overnight at 37 °C. The gel was stained with 0.5% Coomassie Blue for 1 h and incubated with de-staining solution overnight. The degradation of gelatin was observed using Transilluminator 2000 white light (Bio-Rad Laboratories, Hercules, CA, USA). The band densities of MMP2 and MMP9 were measured using Image-J software [36]. For HGF treatment, cells were seeded in 6-well plate overnight, serum-withdrawn for 24 h and treated with 40 ng/mL of HGF alone and with different concentrations (12.5 and 25 µM) of Galangin in 1 mL of serum-free media for 12 h.

### 4.9. Wound Healing Assay

3.5 × 10^5^ cells per well were seeded into a 24-well plate. When the cells reached 100% confluency, they were scratched using a p1000 pipette tip to wound the cell layer. Cell debris were washed with PBS. Then, fresh serum-free medium with 40 ng/mL of HGF and different concentrations (12.5 and 25 µM) of Galangin were added to each well and incubated for 6 h. The wound closure was photographed at 0 and 6 h. The wound recovery was analyzed by Image J (MRI Wound healing Tool) [36]. 

### 4.10. Western Blot Assay

For apoptotic signaling, 1 × 10^6^ CCA cells were seeded in a 60 mm dish for 24 h and treated with various concentrations of Galangin for 48 h. Then, cells were lysed according to the literature [35]. Protein samples of 40 μg were loaded and separated using 12% SDS-PAGE and transferred to a nitrocellulose membrane. The membrane was blocked with 5% bovine serum albumin (BSA) and probed with primary antibodies at 1:1000 dilution. After incubation, the membrane was washed for 10 min in TBS/T 3 times, incubated with secondary antibodies conjugated with horseradish-peroxidase (HRP), and immunoreactive bands were visualized by ECL Plus Western blotting detection system (Bio-Rad Laboratories, Inc., Hercules, CA, USA) using G: Box ChemiXL 1.4 (Syngene; Synoptics, Cambridge, UK). Densitometry analysis was performed using ImageJ software [36].

### 4.11. Proteomics and Data Analysis

To investigate the protein expression profile of different treatment conditions, the extracted proteins were prepared according to previous studies [37]. Briefly, the cells were lysed by RIPA buffer supplemented with protease inhibitor cocktail. Proteins extracted were precipitated using ice-cold acetone and stored at −20 °C for 16 h. After precipitation, the protein pellet was reconstituted in 0.25% RapidGest SF (Waters, UK) in 10 mM Ammonium bicarbonate. A total of 1.2 mg peptides were subjected to LC-MS/MS. The spectrum data were collected in a positive mode on an Orbitrap HF hybrid mass spectrometer combined with an EASY-nLC1000 nano-liquid chromatography (LC) system (Thermo Fisher Scientific, San Jose, CA, USA) with a nano C18 column. The raw mass spectra (.raw file) were processed by Proteome Discoverer 2.4 with identified against the Uniprot protein database (organism: *Homo sapiens*; 20,395 sequences). 

To assemble a differential expressed protein list, multiple consensus workflows were used within the Proteome Discoverer software to compile the peptide-spectrum matches (PSMs) into peptide groups, protein database matches, and non-redundant proteins groups using the principle of strict parsimony as defined by the software defaults. Normalization and differential protein expression analysis was performed on the relative protein abundance matrix conducted using the Bioconductor package ‘DEP’ in R. The cutoff for candidates considered as significantly differentially expressed was LFC of log2(1.5) at a *p*-value less than 0.05. Results were visualized using ‘Enhanced Volcano’ and ‘ggplot2’ packages. Differentially expressed proteins were then analyzed in iLincs SPIA analysis using the KEGG Pathway analysis database for signaling pathway enrichment. The same input was applied for connected perturbagen enrichment in ilincs.org.

### 4.12. Bioinformatics: Drug Target Prediction and Molecular Docking

Putative targets of Galangin were identified based on structural similarity using the SMILES ID as an input in SwissTargetPrediction: http://www.swisstargetprediction.ch/ (accessed on 2 July 2021) [38]. Predicted targets and their relative probability scores were tabulated. Molecular docking was performed using the online tool: SwissDock: http://www.swissdock.ch/ (accessed on 1 August 2021) by inputting the PDB ID or file of the input receptor (c-Met: 1R1W) and ligand (Galangin: 57D). Additionally, the X-ray crystallography structure to compare the binding conformation was queried in Protein Data Bank: (PDB ID: 2RFN). SwissDock provides the various conformations, cluster ranks, binding affinity, and full fitness scores, which were visualized in 3D conformation and analyzed in UCSF Chimera Application (version 1.14). Selected conformation was then saved in a PDB format to be revisualized using BIOVIA Discovery Studio Visualizer tool to generate a 2D interaction plot. 

### 4.13. Statistical Analysis

Data were expressed as mean ± SEM of three independent-experiments and analyzed using Student’s t-test and one-way ANOVA. GraphPad Prism version 7.0 software was used for statistical analysis. Significant value cut-offs were set at * *p*  <  0.05, ** *p*  <  0.01, *** *p*  <  0.001, **** *p*  <  0.0001).

## 5. Conclusions

Galangin is an herbal compound that displayed anti-cancer properties in various cancers. Because Galangin is natural, it is prevalently used in Southeast Asian cuisine and possesses a myriad of biological benefits. Recent developments in identifying novel therapeutic compounds for treating cancer enabled this investigation to unravel anti-metastatic and anti-proliferative properties of Galangin in CCA. Our investigation shows that Galangin effectively inhibits metastatic phenotypes upon HGF stimulation, which indicates the potential of arresting the disease from progressing. Although our investigation shows that Galangin influences the predicted targets, future studies should confirm whether this effect occurs upon binding of the small molecule to the protein or as a collateral effect from a different binding target. While Galangin has proven to be efficacious in a cell line model, the therapy should be further investigated in in vivo models for safety and efficacy and eventually clinical trials as a monotherapy or as an adjuvant therapy

## Data Availability

Data will be provided upon reasonable request.

## Supplementary Materials

The following supporting information can be downloaded at: https://www.mdpi.com/1420-3049/27/14/4664/s1, Table S1: List of Unique Peptides identified in Proteomics; Table S2: Significant Differentially Expressed Proteins; Table S3: Connected Perturbagens; Table S4: SwissTargetPrediction Targets.
